# Proguanil and cycloguanil are organic cation transporter and multidrug and toxin extrusion substrates

**DOI:** 10.1186/s12936-017-2062-y

**Published:** 2017-10-23

**Authors:** Maarten van der Velden, Albert Bilos, Jeroen J. M. W. van den Heuvel, Sanna R. Rijpma, Evelien G. E. Hurkmans, Robert W. Sauerwein, Frans G. M. Russel, Jan B. Koenderink

**Affiliations:** 1 Department of Pharmacology and Toxicology, Radboud University Medical Center, Geert Grooteplein 28, 6525 GA Nijmegen, The Netherlands; 2 Department of Medical Microbiology, Radboud University Medical Center, Geert Grooteplein 28, 6525 GA Nijmegen, The Netherlands

**Keywords:** Proguanil, Cycloguanil, OCT1, OCT2, MATE1, MATE2-K, Anti-malarial, SLC

## Abstract

**Background:**

Malaria, HIV/AIDS, and tuberculosis endemic areas show considerable geographical overlap, leading to incidence of co-infections. This requires treatment with multiple drugs, potentially causing adverse drug–drug interactions (DDIs). As anti-malarials are generally positively charged at physiological pH, they are likely to interact with human organic cation transporters 1 and 2 (OCT1 and OCT2). These transporters are involved in the uptake of drugs into hepatocytes and proximal tubule cells for subsequent metabolic conversion or elimination. This efflux of cationic drugs from hepatocytes and proximal tubule cells into bile and urine can be mediated by multidrug and toxin extrusion 1 and 2-K (MATE1 and MATE2-K) transporters, respectively.

**Methods:**

Here, the interaction of anti-malarials with these transporters was studied in order to predict potential DDIs. Using baculovirus-transduced HEK293 cells transiently expressing human OCT1, OCT2, MATE1 and MATE2K uptake and inhibition was studied by a range of anti-malarials.

**Results:**

Amodiaquine, proguanil, pyrimethamine and quinine were the most potent inhibitors of 4-(4-(dimethylamino)styryl)-*N*-methylpyridinium iodide (ASP) transport, a known substrate of OCT1/2, resulting in half maximal inhibitory concentrations (IC_50_) of 11, 13, 1.6, and 3.4 µM, respectively. Only quinine had a drug–drug index higher than the cut-off value of 0.1 for OCT2, therefore, in vivo pharmacokinetic studies focusing on DDIs involving this compound and other OCT2-interacting drugs are warranted. Furthermore, proguanil appeared to be a substrate of OCT1 and OCT2 with affinities of 8.1 and 9.0 µM, respectively. Additionally, MATE1 and MATE2-K were identified as putative transport proteins for proguanil. Finally, its metabolite cycloguanil was also identified as an OCT1, OCT2, MATE1 and MATE2-K substrate.

**Conclusion:**

Anti-malarials can reduce OCT1 and OCT2 transport activity in vitro. Furthermore, proguanil and cycloguanil were found to be substrates of OCT1, OCT2, MATE1 and MATE2-K, highlighting the importance of these transporters in distribution and excretion. As these compounds shares substrate overlap with metformin DDIs can be anticipated during concurrent treatment.

## Background

In 2015, there were over 200 million cases of malaria, causing more than 400,000 deaths [1]. To treat uncomplicated malaria infections, the World Health Organization (WHO) recommends the use of artemisinin-based combination therapy (ACT), consisting of an artemisinin-derivative and partner drug that has a longer half-life [2]. The use of a variety of different drugs, like anti-malarials taken during pregnancy or for prophylaxis or *Plasmodium vivax* relapse prevention, creates a substantial pool of drugs. Considering the geographical overlap with HIV/AIDS and tuberculosis (TB), co-infections could occur. As there are multiple anti-retrovirals (ARV), anti-TB and anti-malarials available for treatment, there is considerable risk for adverse drug–drug interactions (DDIs) if used concomitantly. These DDIs might lead to under- or overexposure of co-administered drugs potentially causing either treatment failure or toxicity. Although DDIs between current co-treatment of HIV and malaria have been predicted to be low [3], recent studies showed altered anti-malarial plasma concentrations upon ARV treatment [4–6]. Little is known about the potential interaction between anti-malarials and the anti-TB drugs rifampicin and rifabutin [7], although it has been shown that rifampicin increases the clearance of quinine [8].

DDIs frequently occur at the level of biotransformation by cytochrome P450 enzymes in the liver [9], but membrane transport proteins can also be involved [10]. Previously, the potential treatment implications due to possible competition of anti-malarial and antiretroviral DDIs for cellular efflux via ATP binding cassette (ABC) transporters was brought forward [11]. Here, the interaction with organic cation transporters was studied, as anti-malarials are positively charged at physiological pH. Transport proteins belonging to the solute carrier (SLC) family are important for the elimination of drugs, contain members that mainly import cations and have been linked to DDIs. Organic cation transporters (OCT) 1 and OCT2 are cellular uptake transporters belonging to the solute carrier (SLC) family, which are mainly expressed in liver and kidney [12] and within these organs localize to the basolateral membrane of hepatocyte and proximal tubule cells, respectively [13, 14]. Other important SLCs implicated in the transport of cationic drugs are multidrug and toxin extrusion (MATE) protein 1 and MATE2-K. They are primarily located to the apical side of proximal tubule cells (both) [15, 16] and hepatocytes (MATE1) [15], where they export drugs into the urine and bile, respectively. As OCT and MATE transporters are important for the cellular uptake and export of drugs, interaction with these transporters could lead to unwanted adverse effects. Therefore, it is important to address interactions of anti-malarials with these transporters, in order to avoid potential drug–drug interactions in future therapy.

The inhibitory potential of eleven anti-malarials (amodiaquine, artemisinin, atovaquone, chloroquine, dihydroartemisinin, lumefantrine, mefloquine, primaquine, proguanil, pyrimethamine, and quinine) on human OCT1 and OCT2 transport activity was studied using HEK293 cells, in which these transporters were overexpressed by baculovirus transduction. Anti-malarials (25–50 μM) that inhibited transport activity by more than 67% were selected for further determination of their half maximal inhibitory concentration (IC_50_). Furthermore, it was determined which anti-malarials are potential OCT1 or OCT2 substrates and whether MATE1 or MATE2-K could play a role in the cellular efflux of these substrates.

## Methods

### Materials

Human embryonic kidney (HEK293) cells transiently expressing (a combination of) human transporters of interest [organic cation transporter 1 or 2 (OCT1; SLC22A1 or OCT2; SLC22A2), multidrug and toxin extrusion protein 1 or 2-K (MATE1; SLC47A1 or MATE2-K; SLC47A2)], or enhanced yellow fluorescent protein (eYFP) were obtained from PharmTox (Nijmegen, The Netherlands).

BioCoat poly-d-lysine coated 24- and 96-wells plates were purchased from Becton–Dickinson B.V. (Breda, The Netherlands). Dulbecco’s Modified Eagle’s Medium (DMEM) + GlutaMAX-I, Hank’s balanced salt solution (HBSS) and 4-(4-(dimethylamino)styryl)-*N*-methylpyridinium iodide (ASP; ≥ 95% purity) were purchased from Life Technologies Europe B.V. (Bleiswijk, The Netherlands). Fetal bovine serum was purchased from Greiner Bio-One B.V. (Alphen a/d Rijn, The Netherlands). Sodium butyrate, amodiaquine dihydrochloride dehydrate (AQ; analytical standard), artemisinin (ART; 98% purity), atovaquone (ATO; ≥ 98% purity), chloroquine diphosphate (CQ; ≥ 98% purity), dihydroartemisinin (DHA; ≥ 97% purity), lumefantrine (LUM; ≥ 98% purity), mefloquine hydrochloride (MQ; ≥ 98% purity), primaquine diphosphate (PQ; 98% purity), proguanil hydrochloride (PG; ≥ 95% purity), pyrimethamine (PYR; 98.8% purity), quinine (QN; ≥ 98% purity) and 4-(2-hydroxyethyl)-1-piperazineethanesulfonic acid (HEPES) were purchased from Sigma-Aldrich Chemie B.V. (Zwijndrecht, The Netherlands). Cycloguanil (CG; 95% purity) was purchased from Aurum Pharmatech (Franklin Park, NJ, United States). *N*-methyl-quinidine (NMQ) was purchased from Solvo Biotechnology (Szeged, Hungary). Bovine serum albumin fraction V (BSA) was purchased from Roche Diagnostics Nederland B.V. (Almere, The Netherlands). The Victor X3 multimode plate reader was purchased from PerkinElmer Nederland B.V. (Groningen, The Netherlands). Bio-Rad protein assay was purchased from Bio-Rad laboratories Inc. (Veenendaal, The Netherlands). The acquity ultra performance liquid chromatography (UPLC) and Xevo TQ-S micro mass spectrometer was purchased from Waters (Milford, MA, USA). Finally, the HSS T3 analytical column and VanGuard HSS T3 pre-column were purchased from Waters (Dublin, Ireland).

### HEK293 cell culture and transduction

HEK293 cells were modified to transiently express (a combination of) human OCT1, OCT2, MATE1, MATE2-K, or eYFP. Briefly, cDNA of the respective genes was cloned downstream of a CMV promoter into a baculovirus, of which passage three was used to transduce HEK293 cells. These cells were seeded in poly-d-lysine coated 24- or 96-wells plates and grown in a 37 °C incubator at 5% CO_2_. Growth was initiated at ~ 25% confluency using 375 or 125 μL cell and complete medium (DMEM + GlutaMAX™-I supplemented with 10% fetal bovine serum) suspension per well, respectively. After 24 h, cells were transduced with 30 µL virus (10 µL virus + 20 µL complete medium in case of 96-wells plates) or 2 × 15 µL virus for cells that required simultaneous expression of two transporters. Finally, 195 µL sodium butyrate (45 µL for 96-wells plates) was added to a final concentration of 2 mM to enhance protein expression [17].

### Cellular transport inhibition assays

AMO (10 mM), ASP (1 mM), CQ (10 mM), PQ (10 mM) and PG (10 mM) were dissolved in MilliQ water. ART (10 mM), ATO (10 mM), DHA (10 mM), LUM (10 mM), MQ (10 mM), NMQ (20 mM), PYR (10 mM), QN (10 mM) were dissolved in DMSO. All compound stocks were stored at − 20 °C. Inhibition of OCT1- or OCT2-mediated ASP uptake by anti-malarials was performed in poly-d-lysine coated 96-wells plates 2 or 3 days following HEK293 cell transduction. In short, cells were washed with 125 µL 37 °C HBSS containing 10 mM HEPES, pH 7.4. Then, a 50 µL mixture of ASP (10 µM) and anti-malarial compound (25, 50 or 100 µM) in HBSS buffer (pH 7.4) was added to each well and incubated for 10 min at 37 °C. ASP transport was stopped by washing with 125 µL ice-cold HBSS-HEPES buffer supplemented with 0.5% BSA. Following a second wash with 125 µL ice-cold HBSS-HEPES buffer, cells were lysed with 125 µL 1 M NaOH and ASP was excited at a wavelength of 485 nm and fluorescence was detected at an emission wavelength of 535 nm using the Victor X3 multimode plate reader. Anti-malarial inhibition of OCT1- or OCT2-mediated ASP uptake was corrected for background signal (eYFP-transduced cells; mock-transduced), normalized to protein content using the Bio-Rad protein assay and represented as relative ASP uptake after fixing solvent controls to 100%. The mean ± standard error of the mean (SEM) of three independent experiments performed in triplicate were plotted in GraphPad Prism version 5.03 (GraphPad Software, Inc., La Jolla, CA, USA) and significantly reduced ASP uptake was determined using a one-way ANOVA with Dunnett’s post test.

Concentration-dependent inhibition was assessed of anti-malarial compounds that inhibited ASP uptake by more than 67% in the initial screens. For each compound, a range of seven concentrations was used to determine the concentration at which ASP uptake was inhibited half maximally (IC_50_). At least three independent experiments (4 for QN) were performed for each drug in triplicate as described above. Inhibition curves were plotted by nonlinear regression analyses of the data using GraphPad Prism version 5.03. Minimum uptake was set to be greater than 0% and the curve-fitted top within each experiment was adjusted to 100% as follows: each uptake percentage value was multiplied by 100/top in order to let the curve fit start at 100% at the lowest drug concentration. To generate the final dose–response inhibition curve per anti-malarial, percentage of uptake was averaged for each drug concentration per experiment and the resulting mean ± SEM corresponding to three or four independent experiments were plotted.

### DDI index calculation

In order to predict whether future pharmacokinetic studies are recommended to study transporter inhibition in vivo, DDI indices were calculated by using the formula DDI index = fraction unbound * C_max_/IC_50_ with a cut-off value of 0.1 [18]. As C_max_ the (geometric) mean or median maximum plasma concentrations obtained from previous in vivo pharmacokinetic studies after therapeutic dosing was used.

### Cellular import and export assays

HEK293 cells were seeded in 24-wells poly-d-lysine coated plates and transduced the subsequent day with OCT1 or OCT2 as described above. Three days following transduction, OCT1- or OCT2-mediated uptake of ten anti-malarials (AMO, ART, ATO, CQ, DHA, LUM, MQ, PQ, PG, and QN) was performed. NMQ served as a positive substrate for both OCT1 and OCT2 import [19]. In short, cells were washed with 400 µL 37 °C HBSS-HEPES buffer followed by adding 150 µL HBSS buffer with 10 µM anti-malarial or NMQ to each well. After incubation for 15 min at 37 °C, import was stopped by washing with 400 µL ice-cold HBSS-HEPES buffer supplemented with 0.5% BSA. Following a second wash with 400 µL ice-cold HBSS-HEPES buffer, cells were lysed with 200 µL 50% (v/v) MeOH and 0.1% (v/v) formic acid (HCOOH) in water and samples were sent for quantification by mass spectrometry analysis (see “LC–MS/MS quantification of anti-malarials” section). Anti-malarial import mediated by OCT1 or OCT2 was performed in two independent experiments in triplicate per drug and normalized to background (mock-transduced cells). Percentage of anti-malarial uptake (compared to background control) was plotted as mean (6 values) ± SEM in GraphPad Prism version 5.03 and drugs with at least a twofold increase versus background were selected for further analysis.

The affinity (K_M_) and maximum transport rate (V_max_) for OCT1 and OCT2 of PG were determined by analysing concentration-dependent uptake data from three independent experiments. The experimental setup was similar to the cellular import assay, except that a range of four concentrations (2.0, 3.9, 7.8 and 16 µM) and an incubation time of 1 min was used to determine the K_M_ of proguanil. The Michaelis–Menten curve was plotted as mean ± SEM in GraphPad version 5.03 after fixing the V_max_ of each individual experiment to 100% and subtracting mock-transduced background for each concentration used.

Import and export of PG and cycloguanil (CG) was assessed by single and double transduced HEK293 cells. In short, HEK293 cells seeded in 24-wells plates as described, were transduced separately with eYFP, OCT1, OCT2, MATE1 or MATE2-K, or with combinations of these viruses. Following the same procedure as for the cellular import assay described above, the import of 100 µM PG and CG was measured after 5 min incubation and data was plotted as mean pmol/mg/min ± SEM. For export (following OCT1 uptake) of 1 µM PG after 1 min of transport at pH 7.4, percentage of remaining cellular PG (compared to background) was plotted as mean ± SEM in GraphPad version 5.03. Significant reduction in uptake (i.e. export by MATEs) was determined using ln transformation and a one-way repeated measures ANOVA with Dunnet’s post test.

### Liquid chromatography–tandem mass spectrometry (LC–MS/MS) quantification of anti-malarials

Anti-malarial concentration in the cell lysates was quantified using an LC–MS/MS system consisting of a UPLC, a binary solvent manager, a vacuum degasser and an autosampler, coupled to a Xevo TQ-S micro triple quadrupole mass spectrometer. Liquid chromatographic separation of the samples (stored at 10 °C; injection volume of 10 µL) was performed at 40 °C using a HSS T3 analytical column (1.8 μm; 100 × 2.1 mm) coupled to a VanGuard HSS T3 pre-column (1.8 µm; 5 × 2.1 mm). The mobile phase (run time: 10 min) consisted of solvent A [20 mM ammonium formate and 0.5% (v/v) formic acid (HCOOH) in water] and solvent B [0.5% (v/v) formic acid (HCOOH) in acetonitrile] using the following gradient with a flow of 200 µL/min: 0–0.5 min, 50% A; 0.5–4.5 min, 5% A; 4.5–10 min, 50% A. Electrospray ionization (ESI) was operated at a capillary voltage of + 1.0 kV and desolvation and source temperatures of 600 and 150 °C, respectively. Nitrogen was used as desolvation gas with a gas flow of 1000 L/h. Argon was used as collision gas at a pressure of 1.5 mTorr. Positive ion mode was used with selected reaction monitoring (SRM) for the quantitative analysis of AMO, ART, ATO, CQ, CG, DHA, LUM, MQ, PQ, PG, and QN. The peak area of the most abundant product ion was used for quantification. Ions with their corresponding mass/charge (m/z) ratio are shown in Table 1.

**Table 1:** Mass fragments of the most abundant ions for the detection of anti-malarials by LC–MS/MS

Compound name	Precursor ion (m/z)	Product ion (m/z)
Amodiaquine	356.1	283.0
Artemisinin	282.0	162.9
Atovaquone	365.0	337.0
Chloroquine	320.1	247.1
Cycloguanil	252.1	58.0
Dihydroartemisinin	221.0	163.1
Lumefantrine	530.1	512.1
Mefloquine	379.1	361.1
*N*-Methyl-quinidine	339.1	160.0
Primaquine	260.1	243.1
Proguanil	254.0	170.0
Quinine	325.1	307.1

## Results

### Inhibition of OCT1- and OCT2-mediated ASP transport by anti-malarials

**Fig. 1:**
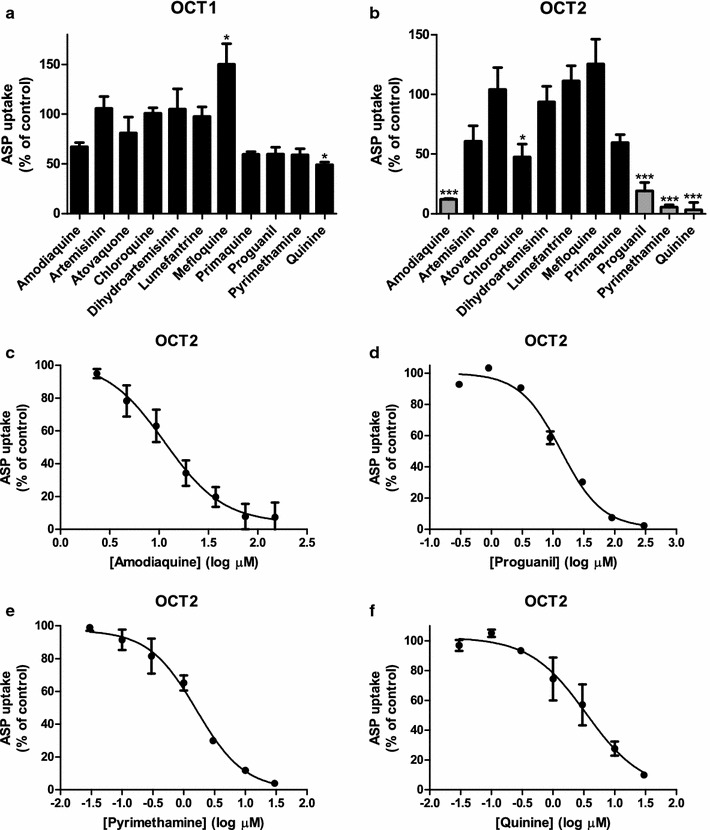
Inhibition of OCT1 and OCT2 by anti-malarials. The inhibition of OCT1 (**a**) and OCT2 (**b**) by eleven anti-malarials (AQ, ART, ATO, CQ, DHA, LUM, MQ, PQ, PG, PM, and QN) at a concentration of 50 μM (except for ATO, LUM: 25 μM, and QN: 100 μM) was studied using 10 μM ASP as a substrate during 10 min incubation at 37 °C. Percentage of ASP uptake was expressed relative to solvent controls, which were fixed at 100%. Anti-malarials showing an inhibitory potential > 67% (grey bars) were selected for IC_50_ determination. Concentration-dependent inhibition of OCT2-mediated ASP (10 μM) uptake during 10 min incubation at 37 °C resulted in IC_50_ values of 11 μM (95% CI 6–22 µM), 13 μM (95% CI 10–17 µM), 1.6 μM (95% CI 1.0–2.5 µM), and 3.4 μM (95% CI 1.1–11 µM) for AQ (**c**), PG (**d**), PM (**e**), and QN (**f**), respectively. *p < 0.05, ***p < 0.001

Using the baculovirus transduction system as described previously [11], OCT1 and OCT2 were transiently expressed in HEK293 cells. These cells were used to study the inhibitory effect of eleven anti-malarials (AMO, ART, ATO, CQ, DHA, LUM, MQ, PQ, PG, PYR, and QN) on OCT1- and OCT2-mediated ASP transport. Uptake of ASP by mock-transduced cells served as a negative control, which was used for background correction. Simultaneous incubation of 10 µM ASP, a known substrate of OCT1 [20] and OCT2 [21], and 50 µM anti-malarial drug (25 µM ATO and LUM, due to precipitation beyond these concentrations; 100 µM QN was used as positive control) for 10 min at 37 °C resulted in different inhibition profiles for OCT1 and OCT2. OCT1-mediated ASP transport in HEK293-transduced cells was only partially inhibited to 49 ± 3% by 100 µM QN (p < 0.05) and even stimulated by MQ to 150 ± 21% (p < 0.05) (Fig. 1a). Four other anti-malarials, AQ, PQ, PG, and PYR inhibited ASP uptake at a concentration of 50 µM for more than 30%, to a relative uptake of 67 ± 4, 59 ± 3, 60 ± 7, and 59 ± 6%, respectively, although these were non-significant inhibitions. In OCT2-transduced HEK293 cells, ASP uptake was reduced to 61 ± 13 and 60 ± 7% by ART and PQ, respectively. AQ, CQ, PG, PYR, and QN significantly reduced ASP uptake to 12.1 ± 0.6% (p < 0.001), 48 ± 11% (p < 0.05), 19 ± 7% (p < 0.001), 6 ± 2% (p < 0.001), and 3 ± 6% (p < 0.001), respectively (Fig. 1b).

The anti-malarials that most potently inhibited OCT1- and/or OCT2-mediated ASP import (more than 67%) were selected for further analysis by determining their IC_50_-values. Using a larger concentration range, inhibition of OCT2-mediated transport of 10 µM ASP by AQ, PG, PYR, and QN was assessed using a similar set-up as above. Sigmoidal inhibition curves were generated by plotting ASP uptake rates as percentage of control versus increasing drug concentrations (Fig. 1c–f). This resulted in the strongest inhibition by PYR and QN with low micromolar IC_50_ concentrations of 1.6 µM (95% CI 1.0–2.5 µM) and 3.4 µM (95% CI 1.1–11 µM), respectively. These were closely followed by IC_50_-concentrations of 11 µM (95% CI 6–22 µM) and 13 µM (95% CI 10–17 µM) for AQ and PG, respectively. In order to assess the clinical relevance of these values, DDI indices were calculated, and a cut-off value of 0.1 was applied to indicate the requirement for future in vivo pharmacokinetic studies [18]. Based on reported C_max_ and unbound fraction values, only QN, with a DDI index of 1.1, fulfilled the criterion (Table 2).

**Table 2:** Calculated DDI indices of the four most potent OCT2 inhibitors

Compound name	C_max_ (ng/mL)^a^	C_max_ (µM)	Unbound fraction	IC_50_ (µM)	DDI index^b^
Amodiaquine	5.2^c^ [45]	0.015	0.1 [46]	11	0.0001
Proguanil	306^d^ [47]	1.2	0.25 [39]	13	0.02
Pyrimethamine	281^e^ [48]	1.1	0.06 [49]	1.6	0.04
Quinine	11430^f^ [50]	35	0.11^g^ [36]	3.4	1.1

^a^(Geometric) mean or median maximum plasma concentrations as determined in in vivo pharmacokinetic studies

^b^DDI index calculated by dividing the unbound C_max_ by the IC_50_ concentration. A DDI index cut-off value ≥ 0.1 is believed to indicate the requirement for future in vivo pharmacokinetic studies [18]

^c^In Ugandan children aged 5–13 years with uncomplicated malaria

^d^In Thai children with uncomplicated multidrug-resistant falciparum malaria

^e^In patients with acute falciparum malaria

^f^In adult patients with uncomplicated falciparum malaria

^g^In Thai patients with falciparum malaria

### OCT1- and OCT2-mediated proguanil and cycloguanil uptake

**Fig. 2:**
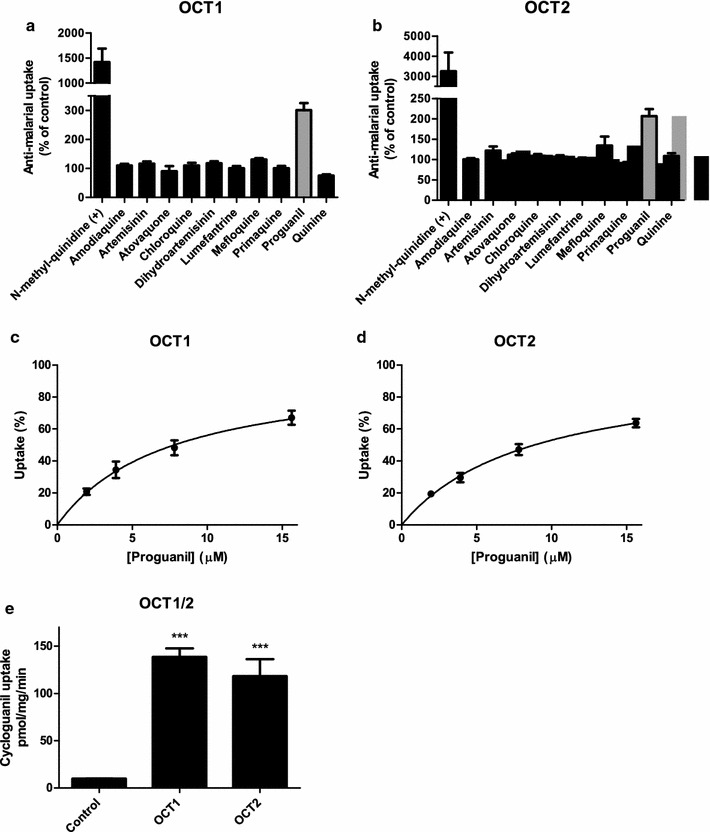
Anti-malarial uptake by OCT1 and OCT2, and uptake characteristics of PG. Uptake of 10 anti-malarials (AQ, ART, ATO, CQ, DHA, LUM, MQ, PQ, PG, and QN) at a concentration of 10 μM by OCT1 (**a**) or OCT2 (**b**) was measured after 15 min of incubation at 37 °C. NMQ served as a positive substrate for both transporters. Uptake was measured in pmol/mg protein/min and expressed as percentage of eYFP control. Anti-malarials showing at least a twofold uptake (grey bars) were selected for further analysis. Transport kinetics of PG during 1 min incubation at 37 °C resulted in a K_M_ of 8.1 ± 1.6 µM and V_max_ of 1840 ± 510 pmol/mg/min for OCT1 (**c**) and a K_M_ of 9.0 ± 1.1 µM and V_max_ of 4440 ± 1500 pmol/mg/min for OCT2 (**d**). Uptake of 100 µM CG for 5 min showed a significantly higher CG uptake for OCT1 (139 ± 9 pmol/mg/min) and OCT2 (118 ± 18 pmol/mg/min) compared to the control (9.7 ± 0.3 pmol/mg/min) (**e**). ***p < 0.001

Next, the potential substrates of OCT1 or OCT2 amongst the tested anti-malarials (AMO, ART, ATO, CQ, DHA, LUM, MQ, PQ, PG, and QN) were identified. Uptake of anti-malarials (10 µM) in HEK293 cells expressing these transporters was determined following 15 min incubation at 37 °C by LC–MS/MS after cell lysis. Uptake was represented as percentage of mock-transduced HEK293 control cells. Anti-malarials with more than twofold increase in uptake of control versus transporter cells were regarded as potential substrates. NMQ was used as a positive OCT1 and OCT2 control substrate. NMQ was taken up by OCT1 and OCT2 as expected. Only one anti-malarial, PG, showed an uptake of more than 200% of the control value. PG was taken up by OCT1 (301 ± 25% of control) as well as OCT2 (207 ± 17%) into HEK293 cells (Fig. 2a, b).

Next, the transport kinetics of PG uptake by OCT1 and OCT2 (eYFP-transduced cells served as background control) were studied. Concentration-dependent uptake of PG was measured during 1 min of incubation at 37 °C and its affinity (K_M_) and maximum transport rate (V_max_) were determined. Uptake of PG by OCT1 was characterized by a K_M_ of 8.1 ± 1.6 µM and V_max_ of 1840 ± 510 pmol/mg protein/min. PG affinity for OCT2 was similar with a K_M_ of 9.0 ± 1.1 µM and V_max_ of 4440 ± 1500 pmol/mg protein/min (Fig. 2c, d).

As PG is metabolized in the liver to its active metabolite CG by CYP2C19 [22], CG transport by OCT1 or OCT2 was investigated. Uptake studies of CG were performed similarly as with the previous anti-malarials, with a 5 min incubation time at 37 °C. This resulted in significant uptake by OCT1 and OCT2 into HEK293 cells (Fig. 2e).

### MATE1 and MATE2-K-mediated proguanil and cycloguanil transport

**Fig. 3:**
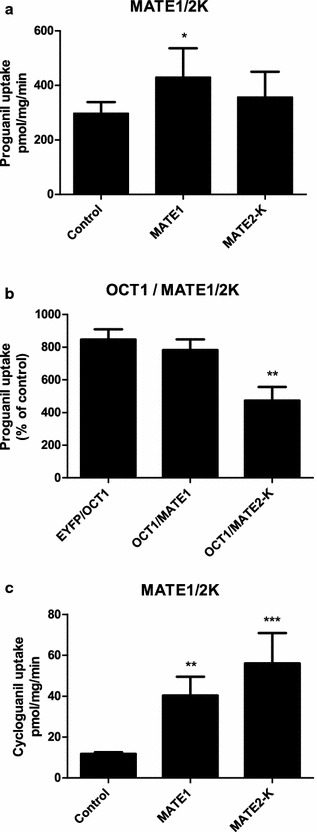
PG and CG uptake by MATE1 and MATE2-K. Uptake of 100 µM PG for 5 min for MATE1 (430 ± 110 pmol/mg/min) and MATE2-K (360 ± 90 pmol/mg/min) compared to the control (300 ± 40 pmol/mg/min) in HEK293 transduced cells (**a**). Following co-transduction of eYFP and OCT1, after measuring uptake of 1 μM PG during 1 min incubation, PG was taken up for 850 ± 60% as compared to eYFP control cells. MATE1 was not capable of reducing OCT1-mediated PG uptake by exporting this anti-malarial, however, MATE2-K reduced PG uptake to 470 ± 80% of control, which is a relative decrease of 44% in uptake (**b**). Uptake of 100 µM CG for 5 min was significantly higher for MATE1 (40 ± 9 pmol/mg/min) and MATE2-K (56 ± 15 pmol/mg/min) compared to the control (11.8 ± 0.9 pmol/mg/min) (**c**). *p < 0.05, **p < 0.01, ***p < 0.001

As PG is likely imported into hepatocytes and proximal tubule cells via OCT1 and OCT2, it was tested whether this compound could be exported by the extrusion transporters MATE1 and MATE2-K. These transporters are able to export and import compounds depending on the physiological conditions. For this purpose, HEK293 cells were transduced with eYFP (mock), MATE1, or MATE2-K. Uptake was represented as percentage of eYFP control cells. Incubation of 100 µM PG for 5 min resulted in small significant uptake by MATE1 (Fig. 3a).

To study PG export capacity of MATE1 and MATE2-K, HEK293 cells were co-transduced with eYFP (mock) and OCT1, OCT1 and MATE1, or OCT1 and MATE2-K. Uptake was represented as percentage of eYFP control cells. Incubation of 1 µM PG for 1 min resulted in eightfold uptake by OCT1 (850 ± 60% of control) (Fig. 3b). This uptake was similar in cells expressing OCT1 and MATE1 (780 ± 70% of control) simultaneously. However, uptake was significantly reduced in cells expressing OCT1 and MATE2-K (470 ± 80% of control, p < 0.01), indicating that approximately 50% of PG was exported by MATE2-K as compared to eYFP/OCT1 transduced cells.

Finally, CG was analysed as a potential MATE1 and MATE2-K substrate by transfecting HEK293 cells with these transporters. Incubation of 100 µM CG for 5 min resulted in significant uptake by MATE1 (40 ± 9 pmol/mg/min) and MATE2-K (56 ± 15 pmol/mg/min) compared to control cells (11.8 ± 0.9 pmol/mg/min) (Fig. 3c). This highlights the involvement of MATE1 and MATE2-K in excretion of PG and its metabolite CG.

## Discussion

In this study, the focus was on the interaction of anti-malarial drugs with the organic cation transporters OCT1, OCT2, MATE1 and MATE2-K. In vitro, OCT1 activity was not affected by any of the tested anti-malarials, while OCT2 was inhibited in the low micromolar range by AQ, PG, PYR and QN. These compounds all share structural features with other OCT2 inhibitors as predicted previously [21].

Inhibition of OCT2 by AQ and PG has not been reported previously. The calculated DDI indices of both drugs were below 0.1, indicating that clinically relevant DDIs related to OCT1 and OCT2 transport are unlikely to occur, but should not be fully excluded as local concentrations at the site of the transporter might be higher compared to systemic levels. Although it has been shown that co-administration of efavirenz (EFV), lopinavir (LPV)/ritonavir (RTV) or atazanavir/RTV with ATO/PG cause a reduction in the AUC of both anti-malarials [4], the decrease in PG level was attributed to induction of CYP2C19 by EFV and LPV/RTV [23, 24]. In contrast, EFV has been shown to increase C_max_ levels of PG by 47%, most likely caused by inhibition instead of induction of CYP2C19 metabolism, which was supported by a reduction in cycloguanil (CG) levels [5]. Another explanation for an increase in PG plasma concentration might be that EFV was also shown to be an inhibitor (absolute IC_50_ of 22 μM) of OCT1 [25] and it was demonstrated that PG is a substrate of OCT1. However, although maximum plasma concentrations of EFV reach 13 μM [26], only about 0.22% is unbound (0.03 μM) [27], resulting in a DDI index for OCT1 well below 0.1, which makes an increase in the C_max_ of PG unlikely. The AUC of EFV has been shown to increase in two subjects in a pharmacokinetic study upon simultaneous AQ/artesunate treatment, resulting in increased hepatic transaminase levels [28]. Although we demonstrated that AQ inhibits OCT2, the calculated DDI index is very low. Hence, boosted EFV levels involving this transporter (and assuming that EFV is an OCT2 substrate) are highly unlikely.

PYR was shown to be the strongest inhibitor of OCT2 activity with an IC_50_ of 1.6 µM. Previously, PYR has been shown to reduce OCT2 activity by 60% at a concentration of 50 µM [29]. This is a substantial difference with the presented findings, but could be explained by differences in cell type (HEK293 vs. HeLa) and substrate (concentration) [ASP vs. metformin (MET)] used. Results from another group, studying inhibition of MET elimination by PYR [30], were more in accordance with ours. Using HEK293 cells stably expressing human OCT2, a K_i_ value of 10 µM PYR for OCT2-mediated MET uptake was found. Although PYR was capable of increasing MET AUC at therapeutic dose, this effect was attributed to inhibition of MATE1 and MATE2-K, as their K_i_ values were over 100-fold lower than the K_i_ value for OCT2 inhibition [30].

In a previous study with stably transfected OCT1 and OCT2 HEK293 cells, inhibition of *N*-[methyl-^3^H]4-phenylpyridinium ([^3^H]MPP^+^) uptake by QN resulted in IC_50_ concentrations of 13 and 23 µM for OCT1 and OCT2, respectively [31]. While QN was found to be more potent at inhibiting OCT1 [31], in this study QN was a more potent inhibitor of OCT2, which might be explained by a different choice of substrate. The presented results more closely resemble two other studies, that also used ASP as a substrate, and found that 20 µM QN inhibited OCT2 by 72% [21] and 100 µM QN inhibited OCT1 by 60% [20]. In the current study, the highest reported DDI index was 1.1 for QN. While DDIs for QN have been described, and a potential role at the transport level by P-glycoprotein/ABCB1 inhibition was studied previously [11], until now clinically relevant interactions caused by QN inhibition of OCT2 have not been reported. A pharmacokinetic study showed that concurrent administration with RTV resulted in a modest increase of C_max_ and AUC of QN [32]. Since RTV is not an OCT2 substrate [33], a transporter interaction with QN is unlikely. Inhibition of CYP2D6 by QN was speculated to cause this effect. In addition, simultaneous RTV and QN administration resulted in an approximately fourfold increase in C_max_ and AUC of QN, which might be due to CYP3A4 interactions [32].

The DDI indices reported here should be interpreted with caution, as multiple factors may influence this value. Firstly, individual peak plasma concentrations in a different population may be substantially higher than the (geometric) mean or median shown in Table 2. Furthermore, the unbound fraction of a drug may alter depending on, e.g. plasma pH [34], pregnancy [35] and disease status. Indeed, in the case of QN treatment, increased intensity of malaria infection correlated with more plasma protein binding (lower unbound fraction) of QN [36, 37]. Still, as QN blood concentration rises with severity of disease [38], unbound QN concentrations may remain at a similar level due to a higher plasma protein binding [37].

**Fig. 4:**
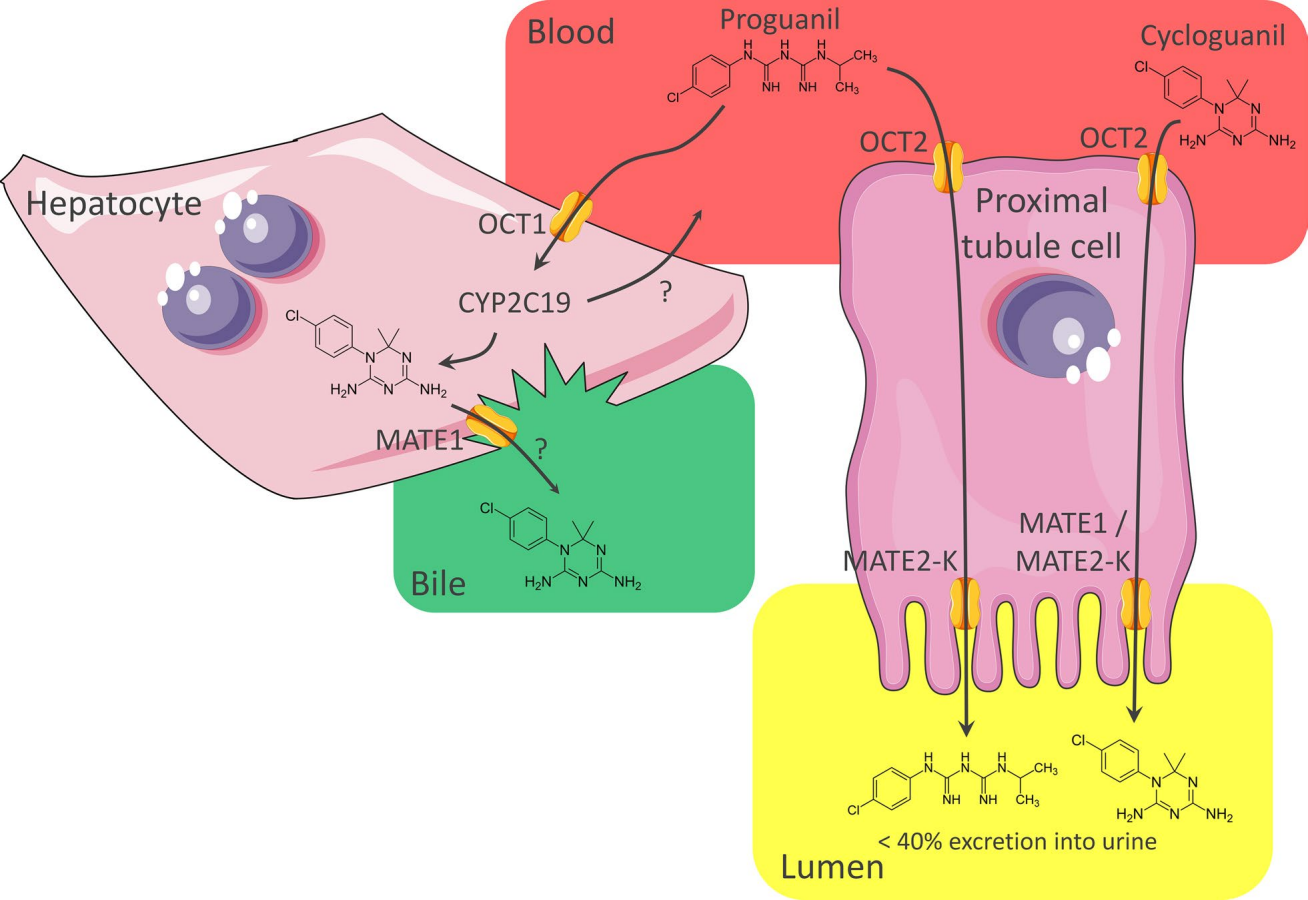
Schematic of proguanil transport and metabolism. Our results suggest PG as substrate of OCT1, which is probably involved in uptake of this compound into hepatocytes, where it is converted to its active metabolite CG by CYP2C19 [22]. We further showed that PG is a substrate of the proximal tubule transporter OCT2 and MATE2-K, which might contribute to the excretion of < 40% unchanged PG into the urine [39]. In addition, CG was suggested to be a substrate of OCT1, OCT2, MATE1, and MATE2-K

PG was found to be the only anti-malarial substrate of OCT1 and OCT2, having similar K_M_ values of 8.1 and 9.0 µM for both transporters, respectively. This highlights OCT1 as a likely liver uptake transporter for PG, where it is metabolized into its active metabolite CG (Fig. 4). Moreover, OCT2 seems to be involved in the extrusion of PG via the kidneys, where it is excreted into the urine for < 40% as well as its metabolites [39]. Similarly, CG also was shown to be a substrate of both OCT1 and OCT2, which requires further investigation. The latter transporter seems to be involved in the renal elimination of CG as this compound can also be excreted into the urine [40].

In addition, when MATE1 and MATE2-K were transduced in HEK293 cells this resulted in a small significant uptake of PG by MATE1. When CG was used as potential substrate, uptake by MATE1 and MATE2-K was clearly present. Furthermore, in order to study the ability of MATE1 and MATE2-K to efflux PG, co-transduction studies were performed. When MATE1 and MATE2-K were co-transduced with OCT1, only MATE2-K reduced the intracellular PG concentration to 56% of its initial value indicating export of PG by this transporter. Thus, both CG and PG are most likely substrates of MATE1 and MATE2-K, who secrete these compounds from the proximal tubule into urine. Interestingly, PG shows structural similarity to MET (both contain guanidine fragments), a known MATE1 [41], MATE2-K [41], OCT1 [42], and OCT2 [43] substrate. It has been shown that polymorphisms in OCT1 can cause reduced liver uptake of MET [44], hence, these variants might also result in reduced hepatic PG uptake leading to less CG conversion. Additionally, as MET is the first-line drug of choice to treat type 2 diabetes (DMII), adverse drug interactions with PG may be anticipated, e.g. when the latter compound is used as prophylactic drug in travelers with DMII.

## Conclusion

In the present study, the interaction of parent compounds with transport proteins was investigated. As part of these anti-malarials undergo biotransformation in the body, their metabolites might also influence transport activity. Therefore, future research should focus on these products as they might increase the DDI potential of their parent compound or have different targets that may cause separate DDIs.

In conclusion, anti-malarials can reduce OCT1 and OCT2 transport activity in vitro. Only QN inhibited OCT2 at a therapeutic relevant dose with a DDI index above the 0.1 cut-off value, warranting in vivo pharmacokinetic studies. However, many factors influence this value and, therefore, individual cases of DDI with other tested compounds cannot be excluded. While anti-malarials and anti-retrovirals are often used concomitantly and may cause adverse DDIs, they most likely are not resulting from interactions with transporters described here due to differential substrate specificity for anti-retrovirals or low DDI indices. Furthermore, CG and PG were found to be substrates of OCT1, OCT2, MATE1 and MATE2-K, highlighting the importance of these transporters in CG and PG distribution and excretion. As PG shares substrate overlap with MET for OCT1, OCT2, MATE1 and MATE2-K, DDIs can be anticipated during concurrent treatment.
